# Supplementary material for: A highly selective and recyclable NO-responsive nanochannel based on a spiroring opening−closing reaction strategy
Source: Nat Commun. 2019 Mar 21;10:1323. doi: 10.1038/s41467-019-09163-4 (PMC6428850; doi:10.1038/s41467-019-09163-4)
Supplement: Supplementary file 1 — Supplementary Information [file 41467_2019_9163_MOESM1_ESM.pdf]

Supplementary Information

A highly selective and recyclable NO-responsive nanochannel based on a spiroring opening-closing reaction strategy

Haibing Li et al

Supplementary Figures

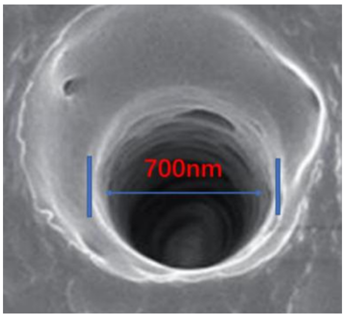

Supplementary Figure 1. SEM image of the bare nanochannel

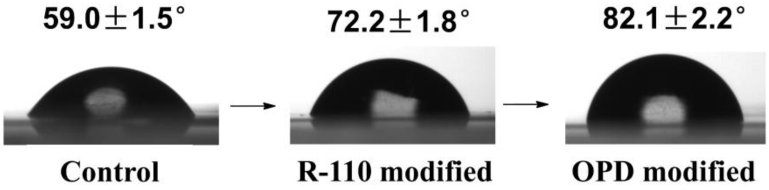

Supplementary Figure 2. Photographs of the water droplet shape on the PET films before and after each step of modification

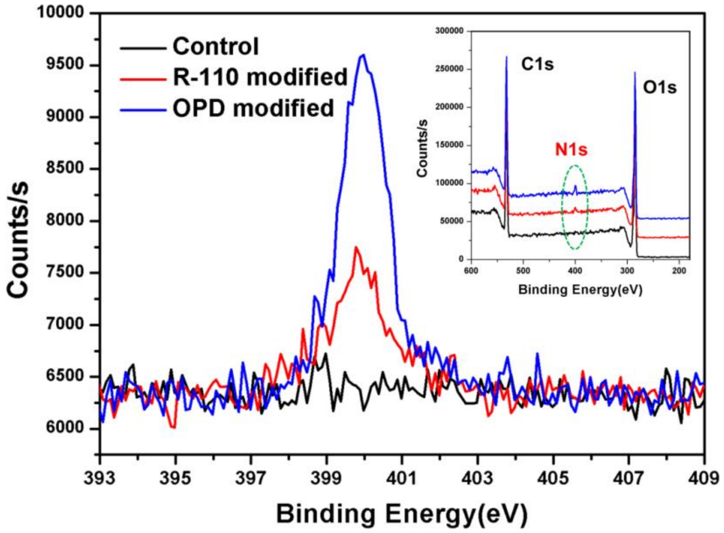

Supplementary Figure 3. XPS of the nanochannel before and after each step of modification

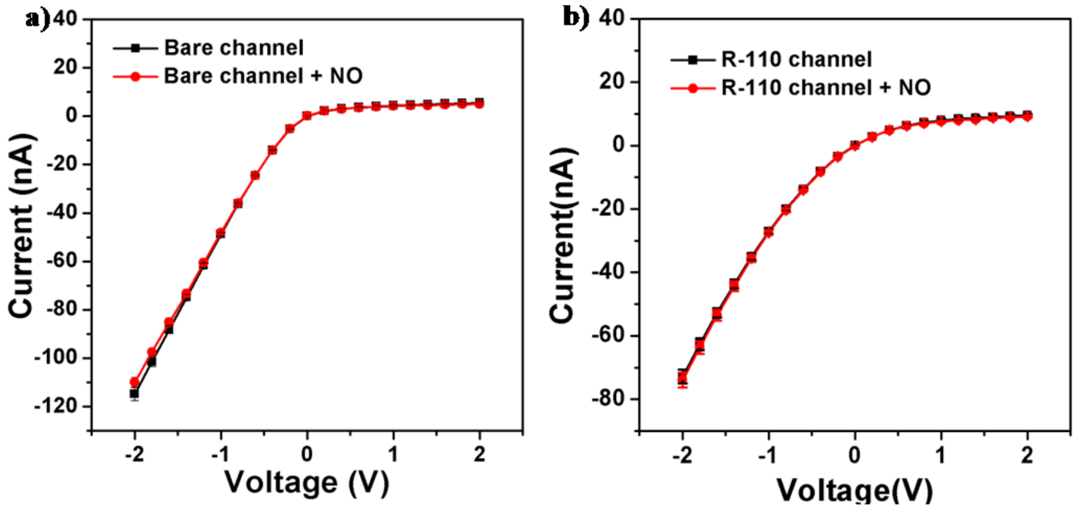

Supplementary Figure 4. (a) I-V responses of the bare nanochannel in the absence and presence of NO; **(b)** I-V responses of the R-110 modified nanochannel in the absence and presence of NO

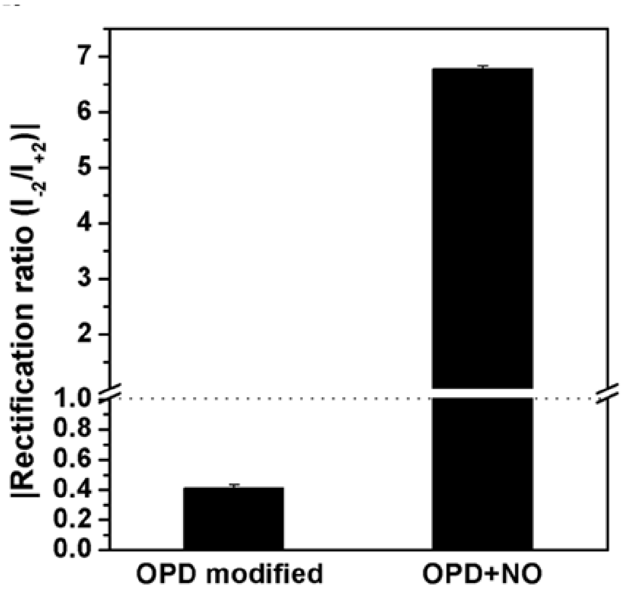

Supplementary Figure 5. The NO-driven ionic rectification ratio show that the OPD modified nanochannels is open by NO

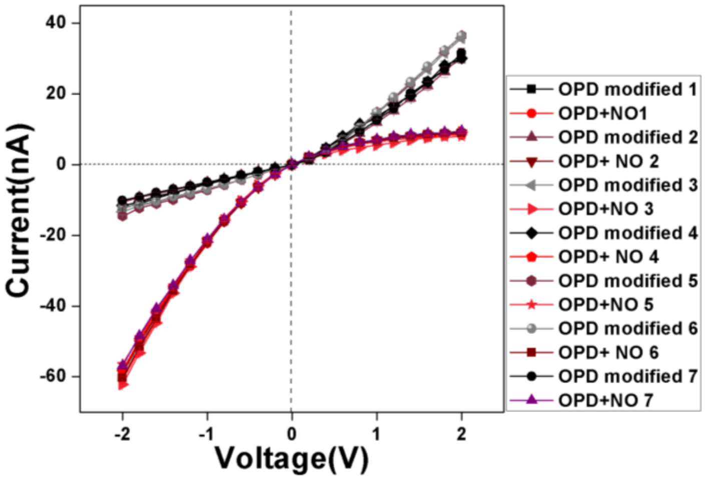

Supplementary Figure 6. The cycling experiments of *I-V* curves in the presence or absence of NO

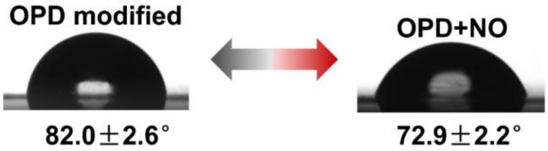

Supplementary Figure 7. The experiments of CA with 10 cycles

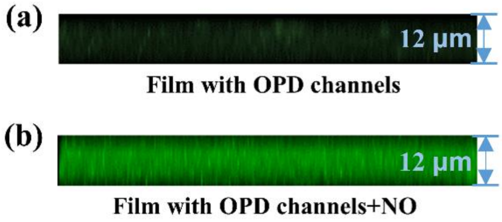

Supplementary Figure8. The cycling experiments of fluorescence signals after 10 cycles, and 12 μm is the thickness of the film

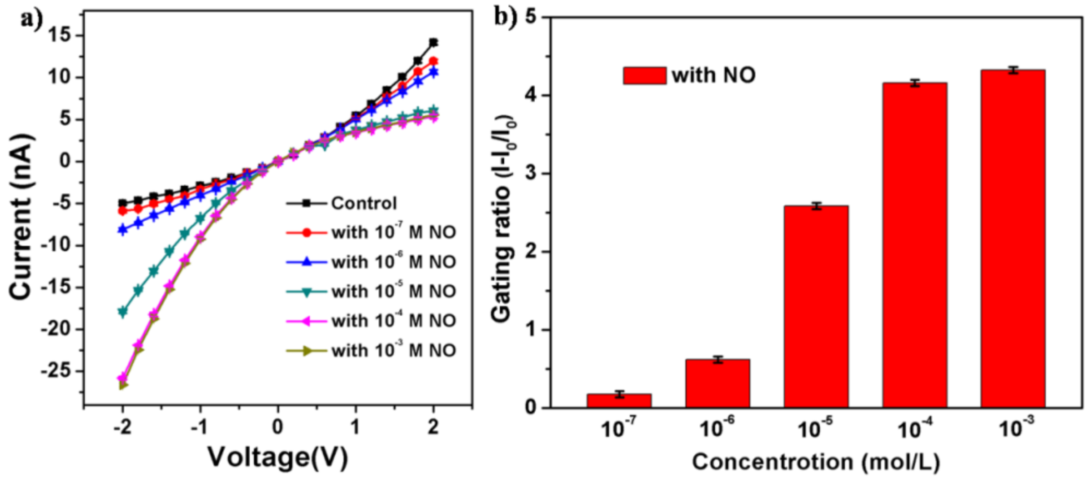

Supplementary Figure 9. *I*-*V* curves and gating ratios obtained in the presence of different concentrations of NO (prepare different concentrations of NO by diluting saturated 2 mM NO solution), with 0.1 M KCl. **(a)** The *I*-*V* curves; **(b)** The gating ratios

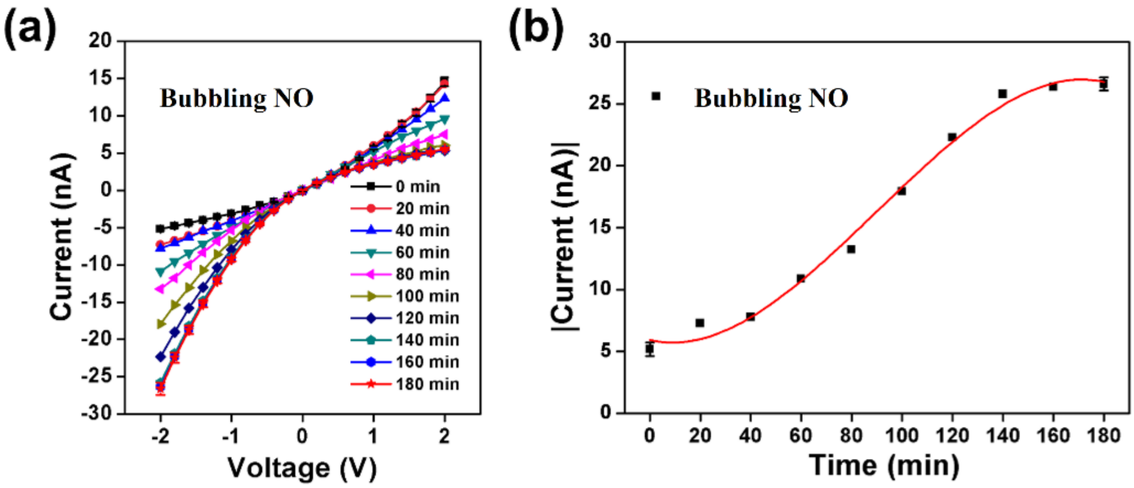

Supplementary Figure 10. (a) The *I*-*V* curves changes with time after bubbing NO; **(b)** The current change at -2V

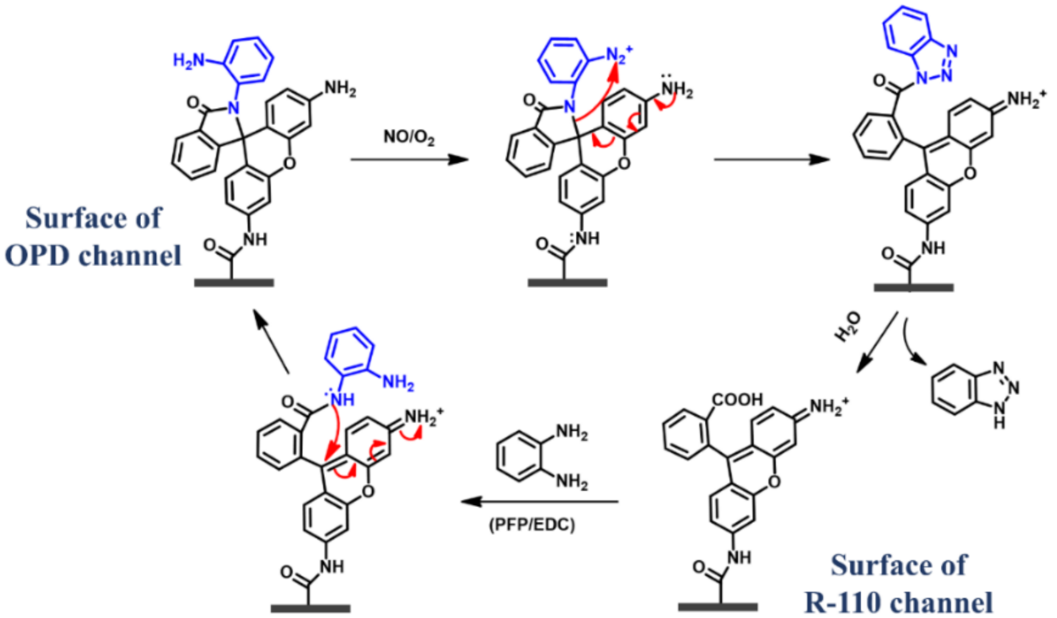

Supplementary Figure 11. The chemical reaction mechanism

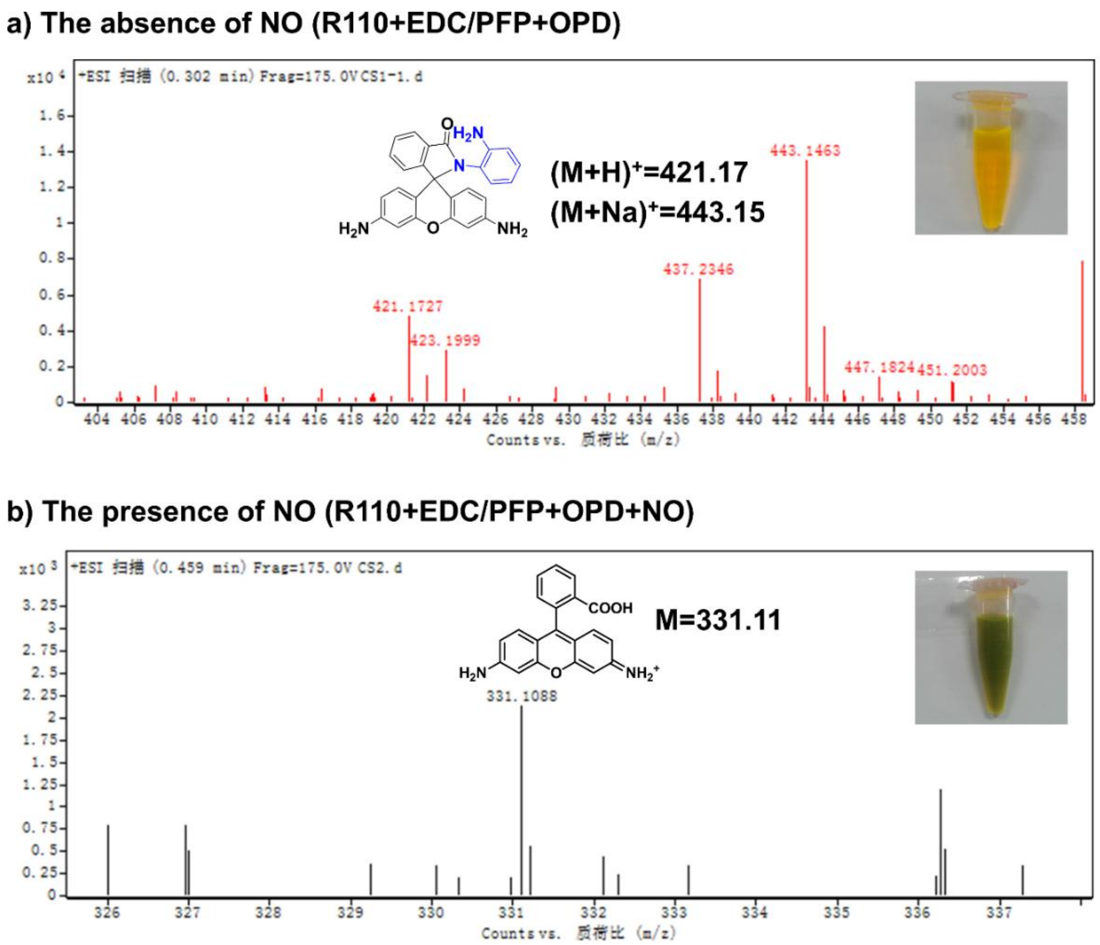

Supplementary Figure 12. The ESI-MS analysis and the fluorescence/color change in the solution. **(a)** in the absence of NO; **(b)** in the presence of NO

a)The ^{1}H NMRin $CDCl_3$(ring closing in the absence of NO)

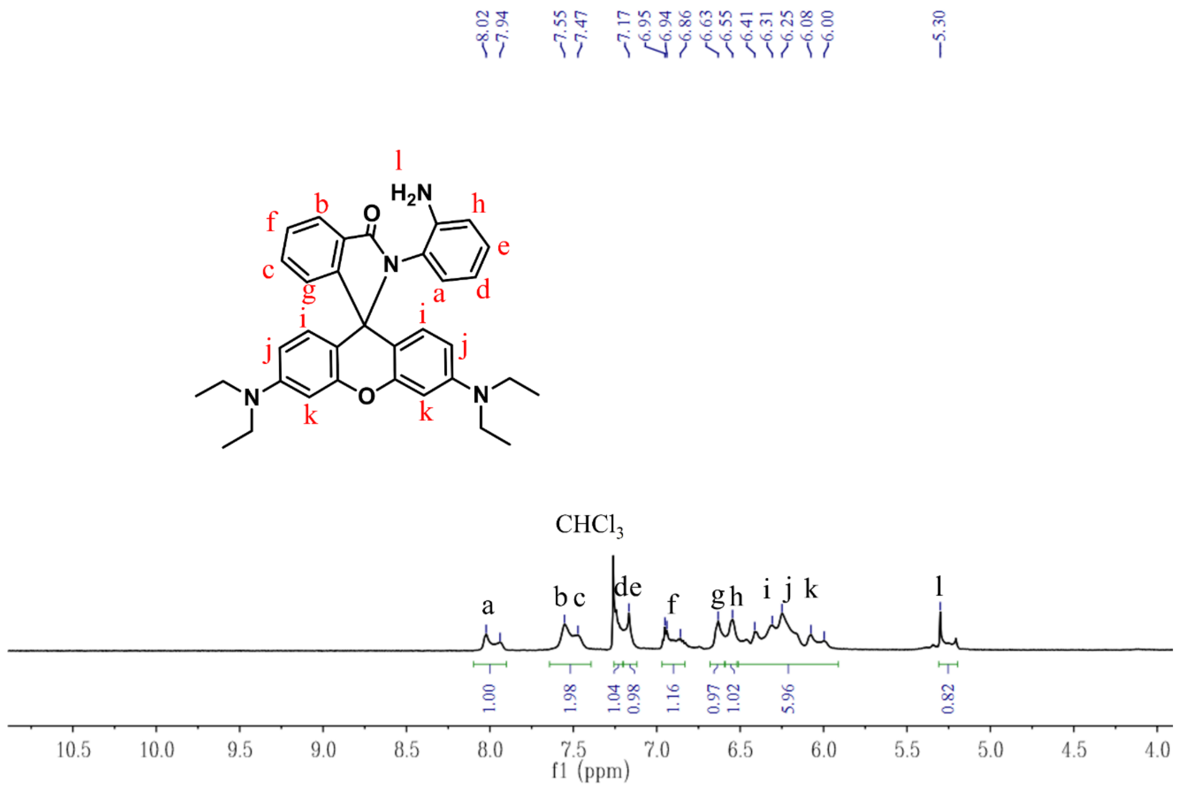

b)The ^{1}H NMR in DMSO(ring opening in the presence of NO)

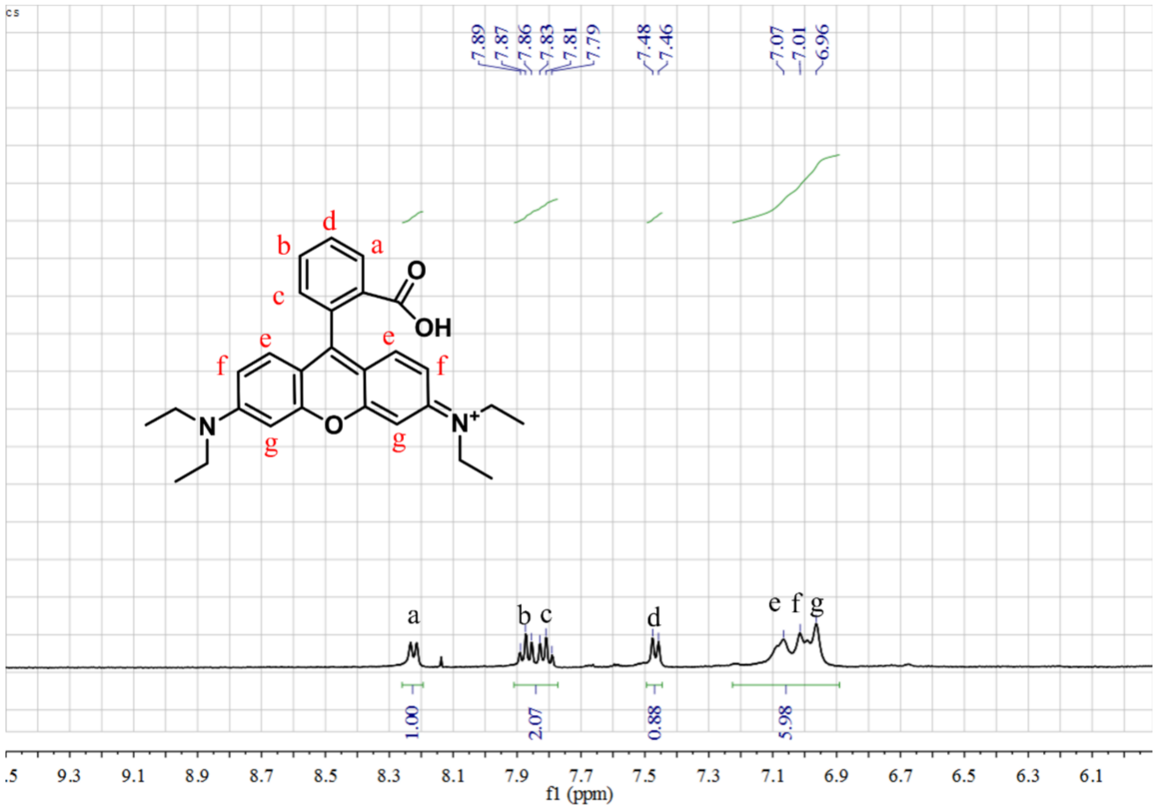

Supplementary Figure 13. The ^{1}HNMR spectrum of spiro ring opening/closing reaction. **(a)** in the absence of NO; **(b)** in the presence of NO

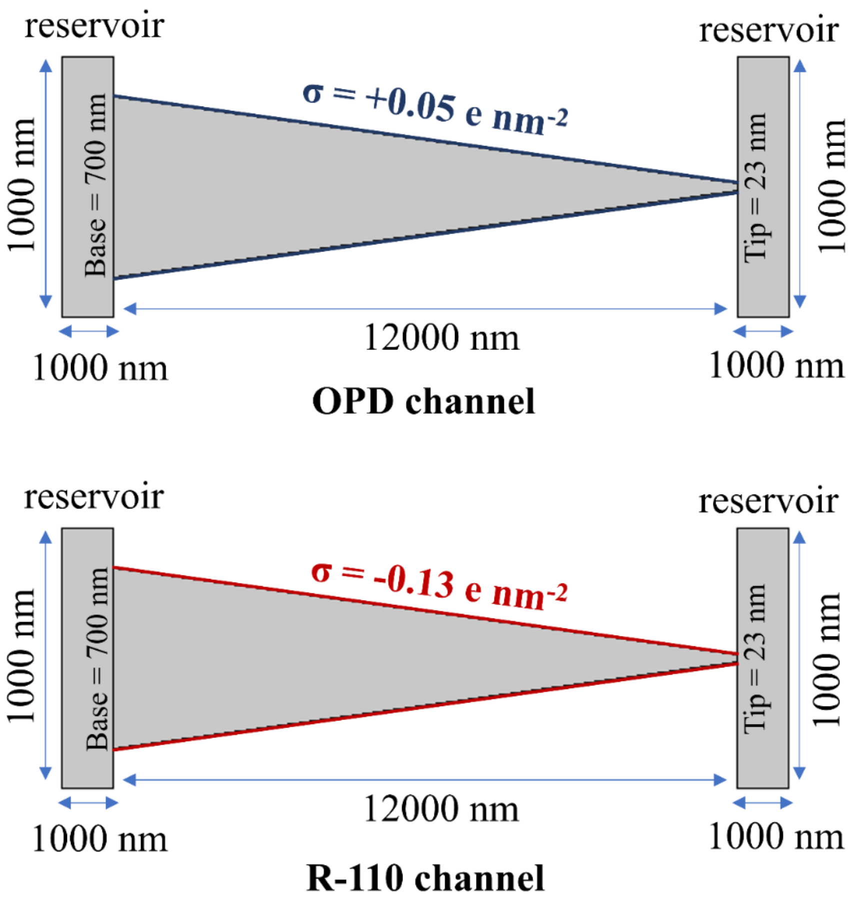

Supplementary Figure 14. The model of numerical simulation about OPD channel and R-110 channel. (length and width are not to scale.) As shown in the Figure, the model of nanochannel is set to 12000 nm (length), 700 nm (base side), and 23 nm (tip side). The reservoirs with a width of 1000 nm and a height of 1000 nm. The surface charge density of OPD channel (+0.05 e nm^{-2}) and OPD channel (-0.13 e nm^{-2}) are measured by EOF experiments. Applying -2V potential at the base side as a driving force to demonstrate the ion behavior under experimental conditions.

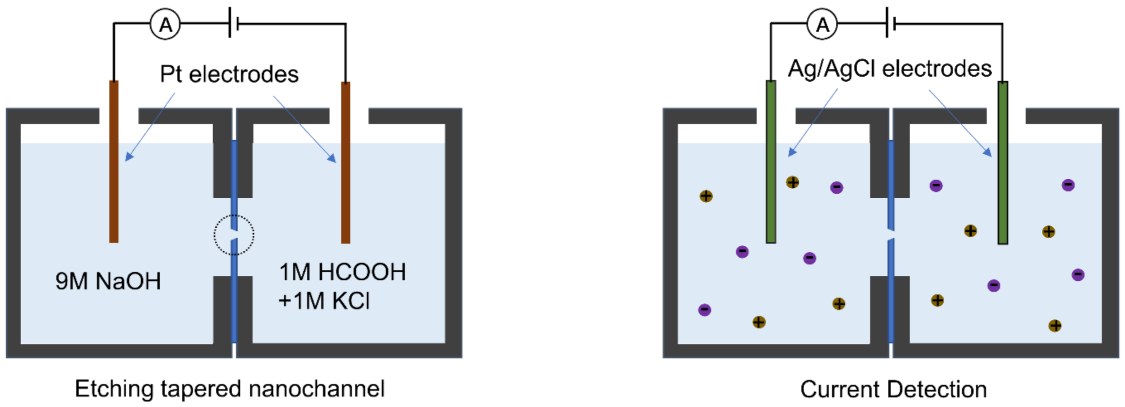

Supplementary Figure 15. Device diagram

Supplementary Table 1. The XPS data of the PET film before modification

Name	Start BE	Peak BE	End BE	Atomic %
C1s	294.03	284.79	280.86	73.95
O1s	537.54	532.93	526.56	26.05

Supplementary Table 2. The XPS data from PET film after R110 modification

Name	Start BE	Peak BE	End BE	Atomic %
C1s	290.88	284.83	281.58	71.93
N1s	404.08	399.90	396.58	**1.42**
O1s	537.08	532.13	528.88	26.65

Supplementary Table 3. The XPS data from PET film after OPD modification

Name	Start BE	Peak BE	End BE	Atomic %
C1s	290.68	284.81	281.78	71.57
N1s	404.08	399.69	396.38	**2.07**
O1s	536.89	532.12	528.68	25.36

Supplementary Table 4. The XPS data from PET film after OPD + NO

Name	Start BE	Peak BE	End BE	Atomic %
C1s	290.68	284.81	281.78	72.83
N1s	403.68	399.69	396.38	**1.74**
O1s	536.98	532.11	528.58	25.43

Supplementary Table 5. surface charge densities for diffrent channels calculated by electroosmotic flow (EOF) experiments

PET membrane	**E**	**V_{eof}(10^{-2} cm s^{-1})**	**σ (e nm^{-2})**
R-110 channels	3.018	2.108	**-0.131**
OPD channels	0.551	-0.811	**0.050**
OPD +10^{-7} M NO	0.627	-0.647	**0.040**
OPD +10^{-6} M NO	0.946	-0.082	**0.005**
OPD +10^{-5} M NO	1.858	1.037	**-0.062**
OPD +10^{-4} M NO	2.770	1.895	**-0.120**
OPD +10^{-3} M NO	3.045	2.131	**-0.130**

Supplementary Methods

Materials. Poly (ethylene terephthalate) (PET, 12 μm thick) membranes were irradiated with single heavy ion (Au) of energy 11.4 MeV/Nucleon at UNILAC linear accelerator (GSI, Darmatadt, Germany). Sodium hydroxide (NaOH), hydrochloric acid (HCl), formic acid (HCOOH), and potassium chloride (KCl) were purchased from Sinopharm Chemical Reagent Shanghai Co., Ltd. (SCRC, China). All solutions were prepared in MilliQ water (18.2 MΩ). Current-voltage curves were measured by a Keithley 6487 picoammeter (Keithley Instruments, Cleveland, OH). Confocal images were acquired using a Zeiss confocal laser scanning unit mounted on a LSM710 fixed-stage upright microscope.

Size of single conical nanochannel

The diameter of large opening of conical nanochannel which was called base (*D*) was determined by scanning electron microscopy (SEM). The diameter of the small opening which was called tip (d_{tip}) was estimated by Supplementary Equation (1):

$$d_{tip} = \frac{4LI}{\pi k(c) U D} \quad (1)$$

L is the length of the pore, which could be approximated to the thickness of the membrane after chemical etching; *I* is the measured ion current; *U* is the applied voltage; d_{tip} and *D* is the tip diameter and the base diameter respectively; *k(c)* is the specific conductivity of the electrolyte. For 1 M KCl solution at 25 °C, *k(c)* is 0.11173 Ω^{-1} cm^{-1}.

SEM Characterization

The diameter of the base was estimated from the multitrack membrane by field-emission scanning electron microscopy (FESEM) which was etched under the same conditions as the single-channel sample. In this work, before modification the base diameter was about 700 nm and tip diameter was estimated by the above relation, tip was about 23 nm.

EOF experiments

The experiment used a porous film with a density of 10^7 cm^{-2}. Tip side of the cell was added with the mixture solution (~ 2 mL) of phenol and electrolyte solution (0.1 M KCl). The other side was added electrolyte solution (as permeated solution). Fluorescence intensity of the permeation solution measured with a fluorescence

spectrometry system every 30 min, and accumulated 150 min. Perform two comparison tests in this way, no voltage applied, and applied voltage.

Phenol, served as an electrically neutral molecular probe, is used for measuring the enhancement factor (*E*). By measuring the fluorescence emission of the phenol in the permeate solution to determine the rate of diffusion. Analogous experiments were completed to determine N_{diff}(the rate of diffusion in the absence of applied current and the N_i (the rate with applied current), Supplementary Equation (2):

$$E = \frac{N_i}{N_{diff}} \quad (2)$$

Peclet number (*Pe*) can be determined by Supplementary Equation (3):

$$E = \frac{Pe}{1-e^{-Pe}} \quad (3)$$

The V_{eof} is determined using Supplementary Equation (4), where *D* is the diffusion coefficient for phenol and D = 8.9 × 10^{-6} cm^2 s^{-1}, *L* is the membrane thickness and L=12 ×10^{-4} cm. Supplementary Equation (4):

$$V_{eof} = \frac{PeD}{L} \quad (4)$$

The ζ potential of the nanochannel walls can be determined by Supplementary Equation (5). Using the known values for the permittivity and viscosity of water (ε = 6.95 × 10^{-10} c^2 J^{-1} m^{-1}, η =0.890 cp; J_{app} is the applied current density; and the measured resistivity of the electrolyte ρ=2.23 KΩ· cm. Supplementary Equation (5):

$$V_{eof} = \frac{-\varepsilon\zeta\rho J_{app}}{\eta} \quad (5)$$

Surface charge density values (σ) can be estimated from the Gouy–Chapman equation (K^{-1}=9.61 × $10^{-9}$$(z^2\ c)^{-1/2}$), Supplementary Equation (6):

$$\sigma = \frac{\varepsilon\zeta}{\kappa^{-1}} \quad (6)$$

Hence, the surface charge density can be calculated from above equation. The calculation results are shown in the Supplementary Table 5.
